# Predictors of Survival in HIV-Infected Patient after Initiation of HAART in Zewditu Memorial Hospital, Addis Ababa, Ethiopia

**DOI:** 10.1155/2014/250913

**Published:** 2014-10-09

**Authors:** Shibre Mengesha, Bekele Belayihun, Abera Kumie

**Affiliations:** ^1^Federal HAPCO, P.O. Box 57101, Ethiopia; ^2^Ethiopian Public Health Association, P.O. Box 7117, Ethiopia; ^3^School of Public Health, College of Health Science, Addis Ababa University, P.O. Box 25819/1000, Ethiopia

## Abstract

*Introduction*. ART has improved the survival of HIV-infected patients. However, patients in resource-poor countries have higher mortality rates, particularly the first months after initiating ART. In this study we tried to determine the survival factors in HIV-infected patients treated with HAART in Zewditu Memorial Hospital. *Methods*. A retrospective cohort study was conducted from 2008 to 2012. All HIV-infected patients above the age of 14 took first line ART. Data were collected, entered, and analyzed using Epi Info 7 and SPSS Version 20. Life table was used to estimate mortality after initiation of ART, and Kaplan-Meier was used to compare survival curves. Cox proportional hazards model was used to assess the predictors of mortality. *Results*. The incidence of mortality was 3.8/100 person-years. Independent predictors of mortality were WHO clinical stages 3-4 (HR = 2.39 at 95% CI (1.26, 5.31)), anemia (hemoglobin level < 10 gm/dL (HR = 5.54 at 95% CI (2.58, 11.86)). *Conclusion*. Incidence of mortality was found relatively low, majority of deaths occurring within 3 months of starting ART. WHO stages 3-4, anemia (hemoglobin count < 10 gm/dL), and past TB coinfection were the main predictors of mortality. The underlying causes for early death in patients presenting at late stages should be investigated.

## 1. Introduction

The revolution in HIV treatment brought about by combination antiretroviral therapy in 1996 had altered the course of the disease among those living with HIV in high-income countries but had only reached a fraction of people in low- and middle-income countries. In resource-poor countries, access to antiretroviral therapy (ART) has improved during the last years and mortality rates among treated patients have declined substantially [1].

Ethiopia is one of the seriously affected countries in sub-Saharan Africa, with more than 1.3 million people living with HIV. In 2003, the Government of Ethiopia introduced its ART program with the goal of reducing HIV-related morbidity and mortality, improving the quality of life of people living with HIV, and mitigating some of the impact of the epidemic [2]. The country has scaled up its ART program and is planning to decentralize the service further to existing health facilities providing ART reaching 517 in December 2009 [3]. In Ethiopia the adult prevalence of HIV was estimated to be 1.5% in 2011 [4]. HAART has provided dramatic reductions in hospitalization and mortality rates. It has also increased the quality of life for many individuals living with HIV [5]. Although some studies demonstrate that the incidence of HIV-related wasting syndrome has also declined in the HAART era, data from the Nutrition for Healthy Living (NFHL) cohort showed that weight loss and wasting are still common in HIV-infected people and that even a 5% weight loss in 6 months markedly increases the risk of death [6]. Nevertheless, mortality has been high compared to high-income countries and factors contributing to this high mortality are poorly understood in resource-limited countries like Ethiopia. However, most of these studies were done in untreated patients. Few studies have evaluated the usefulness of such simple markers as predictors of clinical events in patients receiving highly active antiretroviral therapy (HAART) [6]. In Ethiopia, we treated and followed patients using the WHO clinical stage, hemoglobin level, and the TLC as criteria for beginning treatment [7]. But there is no proper understanding of these factors of paramount importance in tackling the factors of mortality after initiating ART. The aim of this study was to assess predictors of survival rate in HIV patients treated with HAART.

## 2. Methods

### 2.1. Study Design, Period, and Setting

A retrospective cohort study design was carried out from January 2008 to February 2012 at Zewditu Memorial Hospital in Addis Ababa, the capital of Ethiopia. The city area is 450 square kilometers with an estimated population of 2.7 million.

### 2.2. Study Population and Sample Procedures

The study population consists of all HIV-infected patients aged 14 and above who were seen at Zewditu Memorial Hospital HIV Clinic, Addis Ababa. The study participants were PLWH who had been on ART in the hospital from January 2008 to February 2012, who have complete registration, intake, and follow-up forms. Patients who transferred out, lost to follow-up (drop, lost), women who were pregnant at the time of ART initiation, lactating mothers in WHO stage I or II who started ART exclusively to prevent vertical transmission, and patients with competing causes of death other than HIV were excluded. The study subjects were randomly selected based on the inclusion criteria. Profiles of all patients on ART between January 2008 and February 2012 were evaluated, and exposure status was first identified as stages I-II (unexposed) versus stages III-IV (exposed). Finally those who fulfill inclusion criteria were given unique ID number in increasing order for both exposed and unexposed ART groups separately. Then, simple random sampling technique was employed separately to select 416 samples using computer-generated random number table.

### 2.3. Data Collection

Data collection format was developed from ART entry and follow-up form being used in the ART clinic of the Zewditu Memorial Hospital. Data abstraction format was used to collect demographic, laboratory, and clinical data. A total of three days of training was given for data collectors and supervisors. Overall, the principal investigator controlled data collection process. Data quality was ensured by designing proper data collection materials, by checking the collected data daily for completeness, and thorough continuous supervision. All the completed data collection forms were examined again for completeness and consistency during data management, storage, and analysis by principal investigator.

### 2.4. Variables

The main outcome measure was survival rates from the initiation of ART to the end of follow-up date. The independent variables were sociodemographic characteristics, baseline clinical, laboratory, and ART information.

### 2.5. Data Analysis

Data was entered and cleaned using Epi Info Version 7. SPSS Version 20 was used for survival analysis to measure the association of patient's characteristics with time from ART initiation to death. Kaplan-Meier models were used to compare survival times using log rank tests. Cox proportional hazard model was used to identify independent predictors of mortality using hazard ratios with 95% CI. Significant predicator in a bivariate analysis with *P* < 0.05 was included in a multivariable cox regression analysis.

### 2.6. Ethical Considerations

Ethical clearance for the conduct of this study was obtained from the Research and Ethical Committee of School of Public Health of the College of Health Sciences at Addis Ababa University. Permission for data abstraction was sought from Zewditu Hospital. Names or identification numbers of HIV/AIDS patients were not included in the data sheet.

## 3. Results

### 3.1. Sociodemographic and Clinical Characteristics

A total of 416 patients aged 14 years and above, who started ART, were included in this study. From the study participants, 231 (55.5%) of them were males and the mean age was 36.4 (SD = 8.93). Fifty-four percent (54.3%) of the cohort started ART on the late stage of the disease (stages 3-4). The mean hemoglobin level was 12.9 gm/dL (IQR = 11–14). The median CD4 count was 150 cells/*μ*L (IQR = 81–198). The median BMI of patients at the initiation of ART was 22 kg/m^2^. Three hundred ninety-five (97.5%) of the patients had adherence rate of more than 95%. Two hundred twenty three (53.6%) of patients were placed on 3TC-TDF-EFV regimen, 11% of patients had been treated for TB in the past prior to this study, and 96.9% of the patients were not given opportunistic infection prophylaxis. Regarding substance use, 29% (119), 21.4% (88), 8.3% (34), and 33% (137) had been using alcohol, tobacco, hard drug, and soft drug, respectively (Table 1).

### 3.2. Survival Analysis and Cox Regression Analysis

The minimum follow-up time was 0.25 months and the maximum was 43 months. The median follow-up time was 34 months. Thirty-seven (9%) of them were dead. The mean survival time was 39 months. Incidence of mortality was 3.8/100 person-years. The majority 59.5% of deaths occurred within the first 3 months of starting ART.

**Figure 1:**
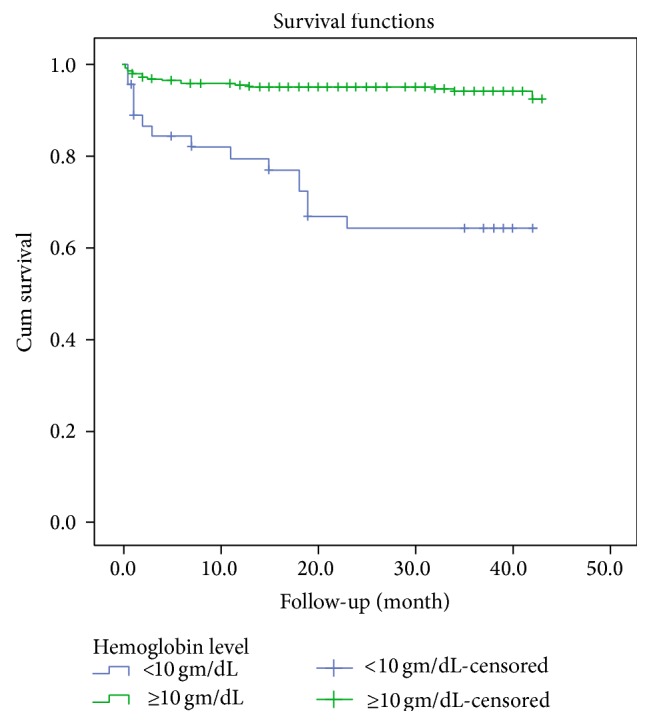
Survival functions of HIV patients by hemoglobin category upon initiation of antiretroviral therapy between 2008 and 2012 in Zewditu Memorial Hospital (*P* < 0.001).

**Figure 2:**
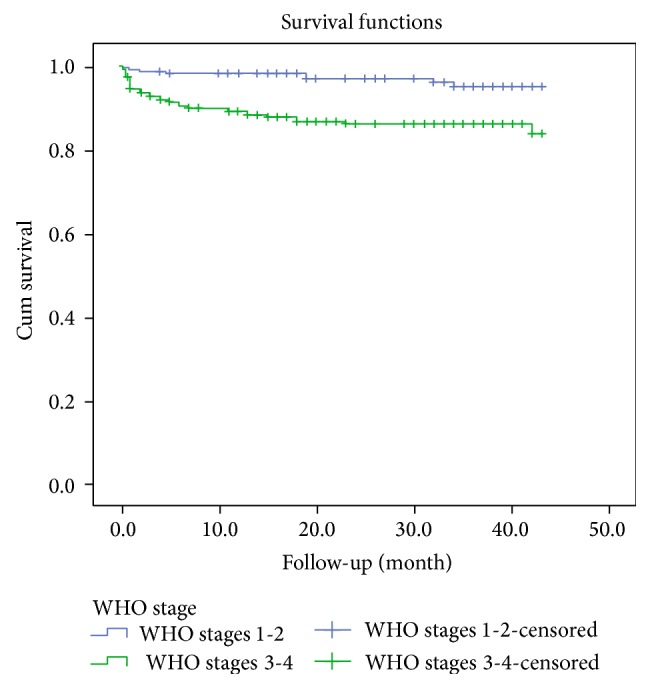
Survival functions of HIV patients by WHO stage category upon initiation of antiretroviral therapy between 2008 and 2012 in Zewditu Memorial Hospital (*P* < 0.001).

**Figure 3:**
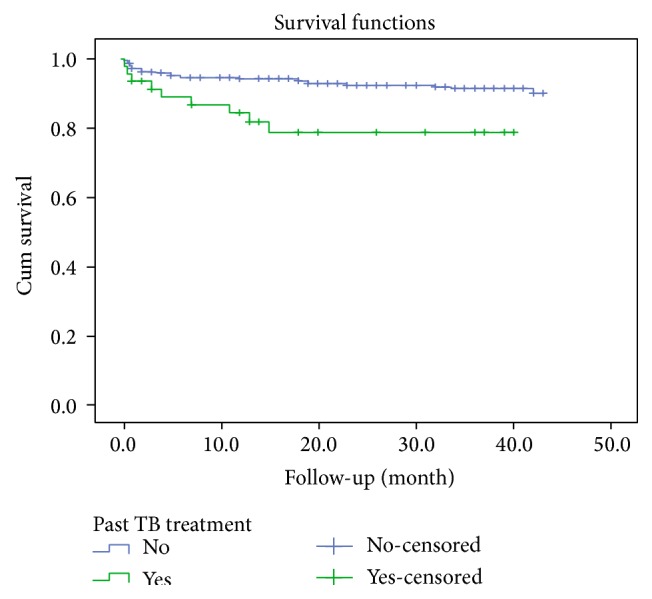
Survival functions of HIV patients by TB coinfection category upon initiation of antiretroviral therapy between 2008 and 2012 in Zewditu Memorial Hospital (*P* < 0.004).

In univariable cox regression analysis WHO clinical stage, anemia and having past TB coinfection were all associated with progression to death. In a multivariable cox regression analysis, significant predictors of mortality were WHO clinical stage (HR = 2.99 at 95% CI (1.26, 5.31)), anemia (HR = 5.54 at 95% CI (2.58, 11.86)), and having past TB coinfection (HR = 4.13 at 95% CI (1.79, 9.51)) (Table 2). Mortality increased with decreasing hemoglobin. The majority of deaths occurred the first three months of the treatment (log rank test, *P* < 0.001) (Figures 1, 2, and 3).

## 4. Discussion

This 5-year retrospective cohort study of AIDS patients on ART gives an insight into survival and its determinants in a hospital setting in Zewditu. The spectrum of patients enrolled in this center was similar for many other start-up ART programs in resource-poor settings. In this cohort, mortality was highest during the first three months of treatment of ART initiation. The WHO clinical stages 3-4, hemoglobin level < 10 gm/dL, and past TB coinfection treatment were the strongest predictor of death in the first month. The findings have practical implications for managing HIV-infected patients in resource-limited settings. The high early mortality in patients with advanced disease implies the need for starting treatment earlier. This requires early diagnosis of HIV infection through improved counseling and testing practices. The majority of deaths occurring during the first three months might be due to the fact that large proportion (54.3%) of the cohort in our study was found to start ART with WHO stages 3 and 4.

The pattern of mortality observed in our cohort is consistent with findings from other resource-poor settings. In Arba Minch Hospital patients, mortality in the first year of follow-up was 15.4/100 person-years and most of deaths occurred within first three months [8].

The incidence mortality rate of 3.8/100 person-years in our cohort is comparable to Arbaminch Hospital cohort in the first year. In Tanzan, a regular decline in mortality from 35.7 during pretreatment follow-up to 17.5 per 100 person-years during the first month of treatment is reported [9]. Another similar study conducted by Johannessen et al. estimated mortality was 19.2, 24.5, and 29.0% with respect to at 3, 6, and 12 months, respectively, with the majority of deaths occurring within three months of starting ART [10]. These findings are similar to what has been reported elsewhere [11, 12].

Anemia was a strong predictor of mortality in our study. Patients with severe anemia had nearly 6 times higher risk of dying during the first year on ART compared to those with a normal hemoglobin level (AHR = 5.54, 95% CI (2.58, 11.86)). Several studies from Africa and Ethiopia have shown that anemia is an independent predictor of mortality in patients on ART, even after controlling for CD4 cell count. Recently, studies from developing countries have found the same association [10, 12, 13]. Indeed, study conducted in Tanzania shows anemia as a strong predictor of mortality; patients with severe anemia had nearly 15 times higher risk of dying during the first year on ART as compared to those with a normal hemoglobin level [10]. Another study conducted in Cameroon indicated that patients with hemoglobin ≤ 8.5 g/dL had two times more risk of death than those with hemoglobin level ≥ 8.5 g/dL [12]. Similar study conducted in Oromia, Ethiopia, showed anemia as predictor of survival [13].

As shown by others, we found that the WHO clinical stage was an independent marker of mortality in patients treated with HAART [12, 14]. Compared to anemia, the WHO stage was a stronger predictor of death in the first month of treatment. While the ultimate goal should be to treat patients before they progress to such advanced stages, doctors in new settings will continue to treat such patients because of poor testing practices in Africa [15]. Therefore, careful documentation of the patient's disease stage will be helpful in identifying patients who need more intense follow-up. Improving the counseling and testing practices should be viewed as a more sustainable strategy. A study conducted in Arba Minch and Malawi WHO clinical stage 4 was important predictor of death [8, 12, 16, 17]. Similarly our results show that WHO clinical stage 4 was main predictor of death.

Koenig et al. found that patients with AIDS who receive a diagnosis of TB during the first months after ART initiation had a mortality rate of 27%, which was 3 times higher than that among other patients [18]. According to Manosuthi et al., patients who delayed ART greater than 6 months after TB diagnosis had higher mortality rate than those who initiated ART less than 6 months after TB diagnosis [19]. Study conducted in Durame Hospital, Ethiopia, showed that patient with positive TB test had 3.9 times more risk than others [20]. Our study also showed similar result; patients having past TB coinfection had 6.5 times more risk than the referent group. We believe that the delay in diagnosis and treatment and not giving TB prophylaxis contributed to the higher mortality.

BMI is described as important prognostic markers either independently or in combination with the TLC both in untreated and in treated patients [8, 16, 17, 19]. Study conducted in rural Malawi showed that individuals who were severely malnourished (BMI < 16 kg/m^2^) had six times higher risk of dying than relatively nourished patients [12]. Another study conducted in Cameroon showed that BMI < 15 kg/m^2^ had three times higher risk of death than BMI > 18.5 kg/m. The rate was two times higher for patients who began ART with a severe immune-depression CD4 < 50 cells/*μ* [7]. This was comparable to what was reported in study done in Tanzania by Johannessen et al. [10]. Study conducted in Durban, South Africa, indicates that CD4 cell count < 50 cell/*µ*L was the most predictor of mortality in HIV patients after they started ART [15]. Our study findings didn't show any association difference which could be due to smaller sample we had in this study but majority of the study participants started ART with CD4 > 50 cell/*µ*L.

## 5. Conclusions 

This study showed that the incidence of death was 3.8/100 person-years with the majority of deaths occurring within 3 months of ART initiation. We found a very high mortality rate in this cohort especially during the three months of treatment. The factors associated with survival were WHO stage, basic haemoglobin, TB coinfection, and anemia. Our study, while confirming the clinical benefit of ART, raises the challenge of earlier and timely access to ART and that of maintaining this clinical benefit over time. This highlights the need for identifying and treating patients early through improved counseling and testing strategies.

## Figures and Tables

**Table 1:** Sociodemographic, clinical, laboratory, substance use, and ART characteristics information of patients who started HHART in Zewditu Memorial Hospital, September 2008–February 2012.

Variable	Categories	Frequency	Mean survival in month	Percent (%)
Sex (*n* = 416)	Male	185	38.9	44.5
	Female	231	40.3	55.5
Age of respondents (*n* = 416)	15–24	18	39.9	4.3
	25–34	181	39	43.5
	35–44	144	39.7	34.6
	>45	73	39.3	17.5
Marital status (*n* = 416)	Single	113	39.6	27.3
	Married	218	39.8	52.7
	Divorced	38	37.9	9.2
	Widowed	45	40.8	10.9
Educational status (*n* = 416)	No education	42	40.5	10.1
	Primary	151	39.6	36.5
	Secondary	155	40.5	37.3
	Tertiary & above	68	38.1	16.3
Religion (*n* = 416)	Protestant	45	39.3	10.8
	Orthodox	348	39.6	83.7
	Muslim	23	40.2	5.5
Cd4 count cell/*µ*L *n* = 393	<50	63	36.9	16
	≥50	330	40.3	84
BMI in kg/m^2^ *n* = 143	<18.5	28	36.9	19.6
	≥18.5	115	40.3	80.4
ART regimen *n* = 416	D4t-3TC-NVP	39	39.2	9.4
	D4T-3TC-EFV	11	32.8	2.6
	AZT-3TC-NVP	49	37.8	11.8
	AZT-3TC-EFV	92	41.2	22.1
	3TC-TDF-EF	225	39.5	54.01
Alcohol use *n* = 408	Nonusers	289	39.2	70.8
	Users	119	39.8	29.2
Tobacco use *n* = 411	Nonusers	323	39.6	78.6
	Users	88	39.1	21.4
Hard drug (iv drug, cocaine,…) use *n* = 412	Nonusers	378	39.6	91.7
	Users	34	39.9	8.3
Soft drug (kchat, shisha) use	Nonusers	278	39.5	67
	Users	137	40.8	33
WHO stage	Stage (1 & 2)	190	41.9	45.7
	Stage (3 & 4)	226	37.9	54.3

**Table 2:** Cox regression analysis by clinical, laboratory, substance use, and ART characteristics information of patients who started HHART in Zewditu Memorial Hospital, September 2008–February 2012 (*n* = 416).

Variables	Alive	Dead	CHR (95% CI)	*P* value	AHR (95% CI)
WHO stage					
1-2	183	7	1.0		1.0
3-4	196	30	**3.89** (**1.71, 8.86**)∗	0.001	**2.99** (**1.26, 5.31**)∗
Hemoglobin in gm/dL					
<10	31	9	**6.55** (**3.27, 13.12**)∗	0.001	**5.54** (**2.58, 11.86**)∗
≥10	285	24	1.0		1.0
CD4 count cells/*µ*L					
<50	54	9	0.52 (0.24, 1.11)	0.09	0.77 (0.33, 1.83)
≥50	306	24	1.0		1.0
BMI in kg/m^2^					
<18.5	26	2	1.13 (0.23, 5.43)	0.882	
≥18.5	108	7	1.0		
Past TB treatment					
No	337	28	1.0		1.0
Yes	38	9	**2.90** (**1.36, 6.17**)∗	0.006	**4.13** (**1.79, 9.51**)∗
Tobacco use					
Nonuser	293	22	1.0		—
User	81	15	0.85 (0.37, 1.93)	0.691	
Soft drug (kchat, shisha) use					
Nonuser	252	26	1.0		—
User	126	11	0.82 (0.41, 1.67)	0.586	
Hard drug (iv drug,…) use					
Nonuser	342	36	1.0	0.235	1.0
User	33	1	0.30 (0.04, 2.19)		0.73 (0.09, 5.65)

A cox proportional hazards model adjusted for all variables listed in the table. HR: hazard ratio; CI: confidence interval; ART: antiretroviral therapy; WHO: World Health Organization; BMI: body mass index.

∗indicates that the variables significantly associated with the outcome at 95% level of significant (*P* < 0.001).
